# Neighbourhood Social Determinants of Health and Geographical Inequalities in Premature Mortality in Taiwan: A Spatiotemporal Approach

**DOI:** 10.3390/ijerph18137091

**Published:** 2021-07-02

**Authors:** Shiue-Shan Weng, Ta-Chien Chan, Pei-Ying Hsu, Shu-Fen Niu

**Affiliations:** 1 Institute of Public Health, National Yang Ming Chiao Tung University, Taipei 112, Taiwan; olisan.weng@gmail.com (S.-S.W.); dachianpig@gmail.com (T.-C.C.); 2 Department of Nursing, College of Nursing, National Yang Ming Chiao Tung University, Taipei 112, Taiwan; 3 Research Center for Humanities and Social Sciences, Academia Sinica, Taipei 115, Taiwan; 4 Department of Health Care Management, National Taipei University of Nursing and Health Sciences, Taipei 112, Taiwan; ericahsu0323@gmail.com; 5 Department of Nursing, Shin Kong Wu Ho-Su Memorial Hospital, Taipei 111, Taiwan; 6 Department of Nursing, Fu Jen Catholic University, Taipei 242, Taiwan

**Keywords:** premature mortality, geographical inequality, neighbourhood, social determinants of health, indigenous peoples

## Abstract

Geographical inequalities in premature mortality and the role of neighbourhood social determinants of health (SDOH) have been less explored. This study aims to assess the geographical inequalities in premature mortality in Taiwan and how neighbourhood SDOH contribute to them and to examine the place-specific associations between neighbourhood SDOH and premature mortality. We used township-level nationwide data for the years 2015 to 2019, including age-standardized premature mortality rates and three upstream SDOH (ethnicity, education, and income). Space-time scan statistics were used to assess the geographical inequality in premature mortality. A geographical and temporal weighted regression was applied to assess spatial heterogeneity and how neighbourhood SDOH contribute to geographic variation in premature mortality. We found geographical inequality in premature mortality to be clearly clustered around mountainous rural and indigenous areas. The association between neighbourhood SDOH and premature mortality was shown to be area-specific. Ethnicity and education could explain nearly 84% variation in premature mortality. After adjusting for neighbourhood SDOH, only a handful of hotspots for premature mortality remained, mainly consisting of rural and indigenous areas in the central-south region of Taiwan. These findings provide empirical evidence for developing locally tailored public health programs for geographical priority areas.

## 1. Introduction

The Sustainable Development Goals (SDGs) for 2030 by the United Nations and previous literature defined premature mortality as deaths from all causes before the age of 70 [1,2]. Premature mortality has been used as a crucial indicator in evaluating a nation’s population health and its health care system’s performance, as it can be prevented through primary prevention or early treatment [3,4]. The SDGs for 2030 include a target to reduce premature deaths in each country by 40% [1]. Therefore, several studies focus on identifying the factors that contribute to premature mortality and quantifying the proportion of deaths that could be avoided if everyone had the same mortality risk [5,6,7,8]. However, a large body of studies on identifying the risk factors for premature mortality ignore the potential spatial variation of mortality, resulting in biased estimation [9,10]. Additionally, in the reality of constrained resources, identifying geographical priority areas to guide decision-making and subsequently intervening and allocating resources to places where people need it the most is essential in reducing health inequality [11]. Specifically, this includes identifying hotspots where people face excess premature mortality risk compared with the rest of the country.

Hotspots or geographical clustering of premature mortality indicates geographical inequality in health. Neighbourhoods may account for such differently place-specific production of health, as they display specific distributions of resources for living and health required in everyday life, such as job opportunities, quality services, and healthy food, which shape and orient people’s behaviours, thus affecting health [12]. Such different contexts of neighbourhoods could indicate that the health of individuals with similar characteristics varies according to the places they live [13]. Therefore, the spatial patterns of health inequalities in premature mortality may relate to the spatial patterns of neighbourhood factors, and the association between premature mortality and neighbourhood factors may vary across neighbourhoods (i.e., spatial non-stationarity). However, no prior studies, to our knowledge, have examined nationwide place-specific associations between neighbourhood factors and premature mortality. 

Furthermore, examining how neighbourhood factors contribute to premature mortality arguably forms a crucial step in generating evidence to support policy development for addressing geographical inequalities in premature mortality. However, few empirical pieces of evidence point to the extent to which geographical inequalities in premature mortality are associated with spatial patterns of neighbourhood factors [2,14]. Two studies based in Ontario, Canada, and Great Britain, using 2011–2015 and 2012–2014 data, respectively, show that two neighbourhood factors—ethnic and socioeconomic status—can explain approximately 80% of geographic variation in premature mortality [2,14]. However, they did not identify whether the distribution of hotspots for premature mortality, after adjusting for these neighbourhood factors, limit implications for policy and research. While several countries have witnessed a downward trend in premature mortality [15,16], inequalities in premature mortality have continued to rise in most European countries [17]. These phenomena reflect the need to consider temporal dependence while assessing geographical inequalities in premature mortality, as well as the role of neighbourhood factors. Recent studies assessing the extent to which spatial patterns of neighbourhood factors are associated with geographical inequalities in premature mortality, have failed to consider temporal dependence, which is the variation in premature mortality across spatial units over time. In some non-Western countries, intra-country variations in premature mortality have also been observed [18,19]; however, most studies only cover the rural/urban or prefecture-divided spatial dimensions of premature mortality. This may mask the heterogeneous characteristics between areas at a lower scale unit, such as townships, and affect the degree of clustering of poor health and place-specific associations between neighbourhood factors and health [20]. An analysis of post-2015 trends, the reference year in SDG targets [21], is lacking. To ensure the timely attainment of this target and to close the inequality gap, it is imperative to understand spatial patterns of premature mortality and the role of neighbourhood factors since the year 2015.

Taiwan is a modern Chinese society; its human development index was 0.91 in 2018, indicating a developed society [22]. The life expectancy is similar to the OECD average (as of 2017, it was 80.4 years for Taiwan, compared with the OECD average of 80.7 years) [4]. However, between 2013 and 2015, Taiwan had an over eight-year disparity in life expectancy between counties with the lowest and highest life expectancy, higher than that of neighbouring non-Western developed countries such as Japan (3.1 years in 2015) and South Korea (6.4 years in 2014) [23,24,25]. This indicates the need to further explore Taiwan’s geographic inequalities. 

In this study, the spatial patterns in neighbourhood factors were focused on township-level social determinants of health (SDOH) because research has pointed towards social factors as the upstream determinants of health [26]. The SDOH may refer to any nonmedical factors influencing health. From the social disadvantage perspective, SDOH include education, income, and race/ethnicity [27]. Socioeconomic factors are known as fundamental drivers of health-related behaviours such as tobacco and alcohol consumption and physical inactivity [26,28], which are also the leading causes of premature mortality [4]. Educational attainment can influence health-related behaviours, the development of social and psychological resources, the abilities necessary to lead healthy lives, and employment opportunities [26,29]. The educational gradient in life expectancy is well-documented in the context of several developed countries [30]. Educational inequalities in premature mortality have also been reported [31,32]. As for income, it reflects the economic resources available for accessing material goods and health-related services [26,29]. The link between income inequality and health has been attributed to the lack of social solidarity [29]. 

Indigenous people are recognized as an ethnic minority but comprise the second large population in Taiwan. During the colonial period in Taiwan, indigenous people were forced to move and settle in concentrated villages along the mountains due to the resettlement policies for keeping them under control, which led to a social network collapse and a loss of land [33]. On this account, 55 townships were proclaimed by law as indigenous areas to protect their rights to land, natural resources, languages, and traditions [33,34]. Most indigenous people choose to live in these indigenous areas, mainly located in central and eastern Taiwan, surrounding dense mountains. In addition to geographic remoteness, Taiwanese indigenous people are also characterized by socioeconomic disadvantages and poorer health outcomes [35], which may increase the risk of geographical inequalities in premature mortality. However, the contribution of neighbourhood indigenous people to geographic variation in premature mortality remains unexplored.

Geographical information systems (GIS) are computer-based tools that can enhance the understanding of the spatial and temporal relationships that affect health risks and outcomes and promote evidence-based decision-making [36]. Therefore, to address the research questions, this study combines the GIS and a spatiotemporal analysis to account for the nonstationary nature of mortality risk over space and time. The following four research questions are put forth. First, are there geographical inequalities in premature mortality in Taiwan? Second, to what extent can geographic variation in premature mortality be explained by spatial patterns in SDOH? Third, what is the distribution of hotspots for premature mortality after the adjustment for the spatial patterns in SDOH? Fourth, is the association between neighbourhood SDOH and premature mortality spatial non-stationary?

## 2. Materials and Methods

### 2.1. Study Design and Setting

A longitudinal ecological study of Taiwan’s population between 2015 and 2019—around 23.5 million individuals per year—was conducted. This used publicly aggregated data based on all townships in Taiwan, except for 19 townships of offshore islands, as spatial autocorrelation cannot be validly presented for offshore islands. Therefore, data in all townships of the main island, consisting of 349 townships within 19 counties/cities, was used. We chose this study period for two reasons: first, 2015 is the reference year for SDG targets; second, this period witnessed a more consistent life expectancy in Taiwan, which could reduce the differences caused by time trends or other unmeasured factors. The average area of a township is 100 km^2^, and the average population density is 1300/km^2^; the average area of a township exceeds 120 km^2^ in mountainous and coastal areas, where the population density is only 340/km^2^ [37]. An institutional review board’s approval was not required for this analysis of publicly available, de-identified, and aggregated data.

### 2.2. Measures 

#### 2.2.1. Age-Standardized Premature Mortality 

Based on the United Nations’ definition in the SDGs for 2030 [1] and previous literature [2], premature mortality is defined as deaths from all causes before the age of 70. The township-level number of deaths by age and township-level population by age were collected for 2015 to 2019 from the Department of Household Registration, Ministry of Interior [38,39]. Premature mortality was standardized by age using the world standard population from 2000 to 2025 [40] through the following calculations:

$$Age-standardized\;premature\;mortality=\frac{\Sigma W_{i}\times A_{i}}{\Sigma W_{i}}$$

where $W_{i}$ is the population in the *i*th age class of the reference population (world standard population from 2000 to 2025), and $A_{i}$ is the age-specific mortality rate per 100,000 people in the *i*th age class (a total of 15 groups covering 0–69 years of age) in Taiwan. Age-standardized premature mortality was used as the dependent variable in this study. 

#### 2.2.2. Neighbourhood Level Social Determinants of Health

We selected three upstream social determinants of health inequalities: ethnicity, education, and income. The townships were considered as the neighbourhoods in this study. Ethnicity was measured using the annual percentages of indigenous people for each neighbourhood (i.e., township) from 2015 to 2019. This was calculated by dividing the number of indigenous residents in each neighbourhood by the neighbourhood-level population. The number of indigenous people in each neighbourhood from 2015 to 2019 was acquired from the Socio-Economic Geographic Information System (SEGIS) maintained by the Department of Statistics, Ministry of the Interior [41]. 

Income was measured using the annual median household income for each neighbourhood from 2015 to 2018. This was obtained from the report of annual individual income tax returns released by the Fiscal Information Agency, Ministry of Finance [42]. As the median household income from 2019 is not yet publicly available, the data for 2019 was replaced with data from 2018. Education was measured using the percentage of people with a college education for each neighbourhood. This was calculated based on data acquired from the SEGIS [41].

### 2.3. Statistical Analysis

The distribution of the age-standardized premature mortality rate in each neighbourhood for the years 2015 to 2019 was presented visually using QGIS 3.4.15 (OSGeo Foundation, Beaverton, OR, USA) [43]. To assess the geographical inequalities in premature mortality in Taiwan for the years 2015 to 2019, space-time scan statistics were calculated using SaTScan software version 9.6 (Information Management Services Inc., Calverton, MD, USA) [44]. This method defines the scanning window as a cylinder with a circular spatial base and a time interval height [45]. The death cases in each neighbourhood for each year, population size of the neighbourhoods for each year, and coordinates of each neighbourhood were utilized in the space–time scan statistics. A likelihood ratio statistic for each circle was computed based on the number of observed and expected cases inside and outside the circle. The coordinates of each township centroid were needed to compute the different sizes of the circular windows. A maximum circle size was set to 5% of the population size to minimize false clusters; this is a size similar to that used in previous studies in Taiwan [46]. The maximum temporal window was three years. The Poisson model was adopted in the SaTScan software, as the number of cases of premature mortality in each neighbourhood in each year followed a Poisson distribution. The test for Poisson distribution was used by *poisson.test* package in R. The window with the maximum likelihood was defined as ‘the most likely cluster’, and the other clusters with statistically significant log-likelihood ratios (LLR) were defined as ‘the secondary potential clusters’. Therefore, if the ‘spatio-time clusters’ of premature mortality were detected, a geographic inequality in premature mortality in Taiwan was considered to exist. 

To explore the extent to which the spatial patterns of the SDOH were associated with geographical variation in premature mortality, a geographical and temporal weighted regression (GTWR), which addresses the data across time and space at a set of locations, was carried out using ArcGIS (ArcMap, version10.3; ESRI Inc., Redlands, CA, USA) with the plugin developed by Huang et al. [47]. A set of local regression coefficients for each neighbourhood were computed using a GTWR model, an extension of the conventional Gaussian geographically weighted regression (Gaussian GWR) model, which adds temporal non-stationarity and employs the spatiotemporal weight matrix, using spatiotemporal distances between observations to measure spatiotemporal relationships [47,48]. A GTWR model fitting for outcome with Poisson distribution has yet to be developed. Therefore, to fit the GTWR model, a log transformation of age-standardized premature mortality rate was carried out to obtain a normal distribution. Thus, GTWR regresses log-transformed, age-standardized, premature mortality based on the percentage of indigenous people, median household income, and percentage of people with a college education. 

To evaluate whether the GTWR model had a better fit than the ordinary least square (OLS) model, we applied a performance comparison between the OLS and GTWR model. An OLS regression was conducted using STATA version 15 [49]. Once again, the dependent variable was the log-transformed age-standardized premature mortality rate (i.e., log-linear model). A residual density plot with kernel-density estimate showed a normal distribution. The effect size from the model was the reported ratio (the exponential of the regression coefficient of the log-transformed data). Adjusted R-square was used to assess whether the GTWR had a better fit to the data than the OLS model. Additionally, the variance inflation factor (VIF) was estimated to detect whether multicollinearity (VIF > 5) was a concern among neighbourhood factors [50]. 

To identify the distribution of hotspots for premature mortality after the adjustment for the spatial patterns in SDOH, a Local Moran’s I cluster map of the residuals for the GTWR was performed visually. The residuals of each area of the GTWR model represent the unexplained spatiotemporal variation in the premature mortality for each area, after adjusting for the neighbourhood SDOH. Previous studies suggest using residuals to understand the extent to which the spatial variation in the outcome declined after adjusting for spatial variables of interest [2,51]. Further spatial clustering of residuals analyses could help identify the clusters for premature mortality after adjusting for the neighbourhood SDOH. The local indicator of spatial autocorrelation (LISA) using Queen’s contiguity spatial weight matrix was performed using ArcGIS 10.3 (ESRI Inc., Redlands, CA, USA), which could yield local Moran’s I for each neighbourhood [52]. In this study, Anselin’s local Moran’s I was used to identify the cluster patterns, because it can identify not only the cluster locations of the cluster but also much more types of spatial clusters than other mapping cluster tools [52,53]. The values of local Moran’s I range from −1 (perfect negative spatial autocorrelation) to +1 (perfect positive spatial autocorrelation) and categorize four types of spatial clusters—high-high clusters, high-low outliers, low-high outliers, and low-low clusters. A high-high cluster indicated that the given township had a correspondingly high residual from the GTWR as its neighbouring townships, the so-called ‘regional hotspot’, and vice versa; a high-low outlier indicated that the township had a higher residual from the GTWR than its neighbouring townships, the so-called ‘individual hotspot’, and vice versa [54]. The Z-value of Moran’s I larger than 1.96 or smaller than −1.96 showed that the null hypothesis of no spatial correlation was rejected. Therefore, if the ‘regional hotspot’ was detected, there would be persistent hotspots for premature mortality after adjusting the spatial patterns in SDOH we explored, suggesting that there were other place-specific factors attributed to this pattern. 

## 3. Results

### 3.1. Geographical Inequalities in Premature Mortality in Taiwan, 2015–2019

**Figure 1:**
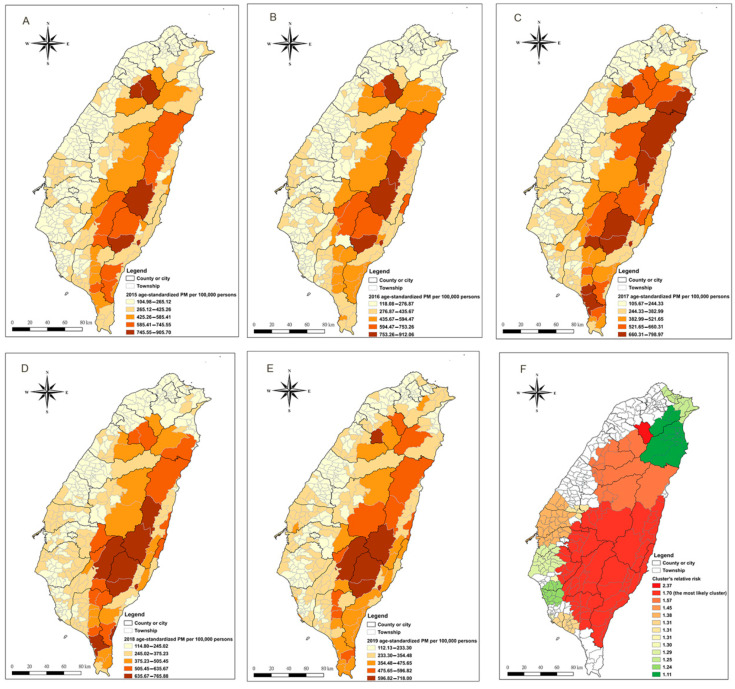
Geographical distribution and spatiotemporal clusters of age-standardized premature mortality. (**A**–**E**) Geographical distribution of age-standardized premature mortality in the township level of Taiwan from 2015 to 2019. (**F**) The significant spatiotemporal clusters of premature mortality from 2015 to 2019. The black line indicates a county or city boundary. The grey line indicates the boundary of a township/neighbourhood within a county or city.

Taiwan is mostly mountainous in the east, with gently sloping plains in the west. As demonstrated from Figure 1A–E, the age-standardized premature mortality has a clear upward trend from west to east (from light orange to brown), regardless of the year. Most townships in the eastern areas had a relatively high age-standardized premature mortality rate between 2015 and 2019 (354.48–912.06 per 100,000 persons), while most townships in the western areas had relatively low rates (104.98–382.99 per 100,000 persons). Most of western Taiwan is urban, and the area has all the special municipalities—the highest-ranking administrative division. East Taiwan mostly comprises rural areas (which is less developed, according to urbanization and stratification of Taiwan townships developed at Taiwan’s National Health Research Institutes) and mountainous areas where indigenous people mainly live. This indicates that premature mortalities in mountainous and rural neighbourhoods were higher than those in plain and urban neighbourhoods. Therefore, spatial heterogeneity in premature mortality was observed in Taiwan, and this geographical disparity in premature mortality has been persistent between 2015 and 2019. For the temporal aspect, a downward trend of the age-standardized premature mortality from 2015 to 2019 was observed. The highest age-standardized premature mortality rate was from 905.7 per 100,000 persons in 2015 to 718.0 per 100,000 persons in 2019.

From the space–time scan statistic results [Figure 1F], 13 significant space–time clusters for premature mortality were identified, indicating that, throughout the study period, the population in these clusters faced higher risks of premature mortality than those outside the clusters. The magnitudes of relative risk for the clusters increased gradually from west to east, which is consistent with the trend of premature mortality rate shown in Figure 1A–E. The most likely cluster (red colour: cluster’s relative risk = 1.70, *p*-value < 0.001) consisted of 64 townships in south-eastern Taiwan located along four of the five nearby main mountain ranges of Taiwan, mostly in rural areas.

### 3.2. Contributions of Neighbourhood Level Social Determinants of Health to Geographical Variation in Premature Mortality

The descriptive statistics for the premature mortality and neighbourhood SDOH in the analyses are listed in Table 1. From Table 2, the OLS model provides global estimates and indicates that the two neighbourhood-level SDOH—ethnicity and educational attainment—were significantly associated with age-standardized premature mortality. The age-standardized premature mortality was found to be positively correlated with the percentage of indigenous people (effect size: 1.01; 95% confidence interval, CI: 1.007–1.008) and negatively correlated with the percentage of people with a college education (effect size: 0.15; 95% CI: 0.13–0.17). The VIF values were all smaller than 5, so multicollinearity was not a concern in this study. Table 3 displays the summary statistics of the local coefficients for every township in the GTWR model. The median effect size revealed that higher premature mortality was associated with a higher percentage of indigenous people and a lower percentage of people with a college education. Therefore, the direction of the median effect size of the GTWR was similar to that of the global estimates from the OLS. In terms of goodness-of-fit, the GTWR model had higher overall adjusted R-square (79.95% for the OLS model and 83.39% for the GTWR model), revealing that the GTWR model fit the data better than the OLS model. The result also showed that the two upstream neighbourhood SDOH—ethnicity and educational attainment—could explain significantly spatiotemporal variation (83.39%) in age-standardized premature mortality in Taiwan.

### 3.3. Place-Specific Associations between Neighbourhood Level Social Determinants of Health and Premature Mortality

**Figure 2:**
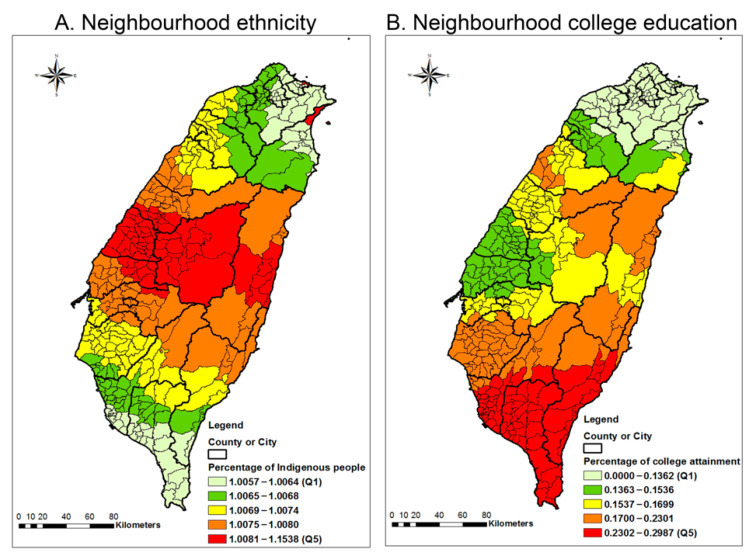
Maps of the GTWR effect sizes for the effect of neighbourhood factors on age-standardized premature mortality. The effect sizes were drawn from a GTWR model that regresses premature mortality on the percentage of Indigenous people, median household income, and percentage of people with a college education. The black line indicates the boundary of a county or city. The grey line indicates the boundary of a township/neighbourhood in a county or city.

The maps of the local effect sizes for the selected significant effect of the two SDOH—ethnicity and educational attainment—on age-standardized premature mortality in each township/neighbourhood allow the visualization of the spatial heterogeneity (Figure 2). Figure 2A illustrates the positive association between the percentage of indigenous people and premature mortality in all the townships of Taiwan. The magnitudes of the local associations between the percentage of indigenous people and premature mortality were higher in the central areas of Taiwan and gradually diminished from the central area outwards. On the other hand, the negative association between neighbourhood college education and premature mortality was high in all townships of Taiwan (Figure 2B). The south-eastern areas generally had a higher effect of neighbourhood college education on premature mortality, and this effect gradually diminished from the south. These graphs (Figure 2A,B) demonstrate that there were spatial variation and non-stationarity in the relationships between the three SDOH and premature mortality.

### 3.4. The Distribution of Hot Spots for Premature Mortality after the Adjustment for the Spatial Patterns in Social Determinants of Health

**Figure 3:**
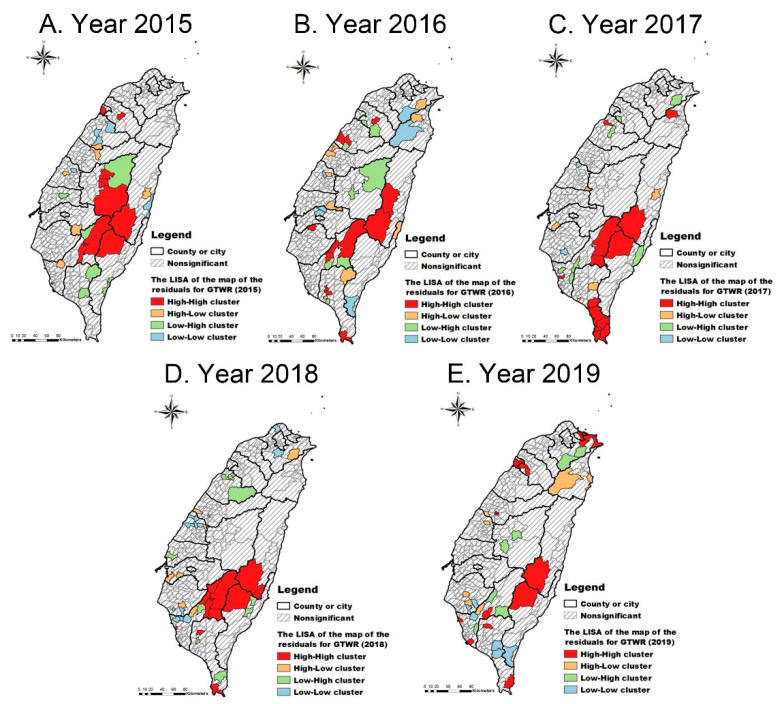
The local indicator of spatial autocorrelation (LISA) cluster maps of the residuals from the GTWR (2015–2019). The black line indicates the boundary of a county or city. The grey line indicates the boundary of a township/neighbourhood in a county or city. The grey slashes indicate no cluster pattern of the residuals.

To explore the distribution of clusters for premature mortality after the adjustment for the spatial patterns in the SDOH we explored, a map of local Moran’s I of residuals for GTWR was conducted and is presented in Figure 3. In these maps (Figure 3A–E), after the adjustment for the neighbourhood SDOH, the hotspots for premature mortality decreased significantly, with only a handful of townships/neighbourhoods left, which were mainly rural and indigenous areas in the central-south region of Taiwan. This result indicated that some variations in those neighbourhoods could not be explained by the three neighbourhood SDOH—ethnicity, education, and income.

## 4. Discussion

This study used nationwide data from 2015 to 2019 and incorporated spatial–temporal dependence among neighbourhoods to assess the geographical inequalities in premature mortality and evaluate the role played by the spatial patterns of SDOH (ethnicity, education, and income) in Taiwan. We found a downward trend of premature mortality from 2015 to 2019, revealing the progress toward reducing premature mortality in Taiwan. However, the geographical inequalities in premature mortality persistently exist. We found a clear upward trend of premature mortality rate from plains to mountain ranges (i.e., from west Taiwan to east Taiwan). The highest premature mortality was clustered surrounding the densely mountainous area of Taiwan (i.e., neighbourhoods in south-eastern areas). The spatial patterns of SDOH (i.e., ethnicity and education) made large contributions to the geographical variation in premature mortality. After the adjustment for such upstream factors, the hotspots for premature mortality decreased significantly—a handful of neighbourhoods having hotspots for premature mortality. These results give empirical evidence to support that SDOH are upstream factors for premature mortality.

The location of the clusters of premature mortality was along nearby mountain ranges of south-eastern areas, a mostly rural mountainous region. This indicated that premature mortality was likely to diffuse across administrative boundaries of local neighbourhoods. Although rural–urban differences in premature mortality varied by countries and time, most of countries were found to have higher rates of premature mortality in rural than urban areas [2,14,18,55]. Rural areas have historically faced constraints related to achieving good health outcomes, including lower opportunities of employment and education, barriers to health care access, less comprehensive health-care resources, higher transportation costs, and less proximity to supermarkets [56,57,58,59,60]. Furthermore, the highest clustering of premature mortality we found was situated exactly along four of the five main mountain ranges in Taiwan (including the Coastal Mountains Range, Central Mountains Range, Yushan, and Alishan Range) and nearby areas. Such natural surroundings created comparative geographic isolation and exacerbated the constraints of rural areas. This may be one of the plausible explanations for why clusters of premature mortality in other rural areas of Taiwan were not found. Therefore, the densely mountainous geographical features of Taiwan may have created geographical inequalities in health. 

This study showed that geographical variation in premature mortality in Taiwan was largely explained by ethnicity and education—the indicators of social disadvantage [27]. This finding was consistent with previous studies [2,14]. After taking spatial non-stationarity association between those factors and premature mortality into consideration, the hotspots for premature mortality decreased significantly. This provided evidence that social disadvantage is a significant associated factor for geographical inequality in premature mortality. Universal health care insurance has been viewed as an important measure to tackle health inequality [61,62,63]. Taiwan has launched a universal compulsory national health insurance (NHI) program that has covered 99.6% of its residents since 1995 [64]. NHI has several properties that can improve the accessibility and affordability of health care, including the absence of gatekeepers (generally, people can go to clinics/hospitals without requiring permission), short waiting periods, low cost of co-payment and insurance, and mobile health services for remote areas [65,66]. However, our findings showed that geographical inequalities in premature mortality of Taiwan persistently exist, although the rate of premature mortality has decreased in recent years. This echoes the argument that health care is necessary but not sufficient to reduce health inequalities [67]. In addition, the remaining clusters after the consideration of the three neighbourhood SDOH revealed that the variations of premature mortality in those neighbourhoods were not completely explained by the variables investigated. Additional work is required to investigate the place-specific contextual factors that may attribute to this pattern within these neighbourhoods.

In all, the two neighbourhood-level SDOH—ethnicity and education—were significant and found to vary across space for the premature mortality in Taiwan. This highlights the need to consider the spatial non-stationary relationships between SDOH and premature mortality to have a more accurate estimation. This study also suggests that, among the three neighbourhood SDOH, the neighbourhood level of education attainment is the strongest predictor of premature mortality. This may be because education shapes employment opportunities, health behaviours, and other SDOH [68]. This could also explain why we could not establish a statistically significant relationship between neighbourhood income and premature mortality. Another explanation may be that Taiwan has relative income equality, compared to neighbouring non-Western countries, the United Kingdom, and the United States of America [69], and the link between income and health inequality in health was mainly a result of income inequality [29]. Therefore, increased effort on the investment in education for those hotspots for premature mortality is imperative, especially for southern areas of Taiwan, which are significantly affected by education inequalities.

The neighbourhood percentage of indigenous people is another significant contribution to the geographical inequalities in premature mortality. We found that the spatiotemporal clustering of the highest premature mortality rates in Taiwan (i.e., neighbourhoods in south-eastern areas) incorporates not only rural mountainous areas, but also indigenous areas. These findings indicate that, in Taiwan, existing social welfare policies for indigenous people, such as household financial support and mobile health services, have a limited effect on narrowing health inequalities. The issues of historical trauma, discrimination based on ethnic identity, and lack of cultural competence in health care have been considered as the root causes for poorer health outcomes among indigenous people [70,71,72]; however, interventions addressing these issues are rare. Therefore, to ensure the timely attainment of SDG targets and to close the inequality gap, reform of current policies and health programs for indigenous people is needed. Moreover, Taiwanese indigenous people mainly live in socioeconomically deprived neighbourhoods, which has exacerbated the socioeconomic inequalities and subsequent geographical inequalities in premature mortality between indigenous people and their non-indigenous counterparts. Based on the finding that geographical inequalities in premature mortality are significantly affected by education, the allocation of education resources to indigenous areas is important for narrowing geographical inequalities in premature mortality.

A limitation of this study was that we could not obtain information about the township-level premature mortality by specific causes. Although the main causes of death across townships in Taiwan are similar, mostly due to non-communicable diseases, premature deaths from accidents and suicides may reflect geographic physical and psychosocial disparities. These factors, therefore, merit further attention. Moreover, as an ecological study, the findings of this study could not prove causation, and this limits the individual level inferences. While we failed to obtain individual data, data from the township level is the most optimal geographic unit. Finally, although we chose upstream independent variables—neighbourhood ethnicity, income, and educational attainment—under the consideration of the spatiotemporal effect, it cannot be denied that our results are likely explained by a potential confounder, which can influence both the independent and dependent variables and cannot be a mediator—for example, residential self-selection bias. However, whether it can explain the association between neighbourhood ethnicity, income, and educational attainment and premature mortality is unclear. A previous study found that residential self-selection was not a major source of bias in the relationship between neighbourhood socioeconomic factors and physical activity or body mass index [73].

## 5. Conclusions

This study demonstrated that there were geographical inequalities in premature mortality in Taiwan. The clustering of the highest premature mortality was mainly located in mountainous rural areas and indigenous areas. Neighbourhood SDOH—ethnicity and education—can considerably explain such geographical variation in premature mortality. Only a handful of neighbourhoods with hotspots for premature mortality remained after adjusting for these neighbourhood SDOH. These findings provide empirical evidence that neighbourhood SDOH or social disadvantage is a significant predictor of the geographical inequalities in premature mortality. This study also presented the varying spatial impacts of neighbourhood SDOH on premature mortality. Therefore, public health action and national programmes need to prioritize interventions that improve neighbourhood SDOH—upstream factors—in mountainous indigenous communities with the most likely clusters of premature mortality. 

## Figures and Tables

**Table 1:** Descriptive statistics of premature mortality and neighbourhood SDOH (*n* = 349).

Characteristics	Median (25th, 75th Percentile)
Age-standardized premature mortality rate (per 100,000 persons)	231.11 (193.8, 282.8)
Neighbourhood ethnicity	
Percentage of Indigenous people (%)	1 (0, 2)
Neighbourhood socioeconomic status	
Median household income (NT$1000)	575 (544, 613)
Percentage of people with a college education (%)	34.3 (27.7, 42.9)

Note. NT$ = New Taiwan dollar.

**Table 2:** Log-transformed age-standardized premature mortality regressed on neighbourhood SDOH using ordinary least squares model (*n* = 349).

Characteristics	Effect Size	95% CI	*p*-Value	VIF
Neighbourhood ethnicity				
Percentage of Indigenous people	1.01	(1.007–1.008)	<0.001	1.35
Neighbourhood socioeconomic status				
Median household income	1.00	(0.99–1.00)	0.168	3.04
Percentage of people with a college education	0.15	(0.13–0.17)	<0.001	3.55
Adjusted R-square	79.95%			

Note. Effect size was computed by the exponential of the regression coefficient; CI = confidence interval; VIF = variance inflation factor.

**Table 3:** Log-transformed age-standardized premature mortality regressed on neighbourhood SDOH using geographically and temporally weighted regression (*n* = 349).

Characteristics	First Quartile	Median	Third Quartile	95% CI
Neighbourhood ethnicity				
Percentage of Indigenous people	1.01	1.01	1.01	1.0075–1.0079
Neighbourhood socioeconomic status				
Median household income	0.99	1.00	1.00	1.0000–1.0002
Percentage of people with a college education	0.15	0.17	0.21	0.17–0.18
Adjusted R-square	83.39%			

Note. Effect size was computed by the exponential of the regression coefficient; CI = confidence interval.

## Data Availability

Publicly available datasets were analysed in this study. This data can be found here: (1) The township-level number of deaths by age: https://data.moi.gov.tw/MoiOD/Data/DataDetail.aspx?oid=C26578D2-2DD8-4E1C-A03C- (2) Statistical Yearbook of Interior: https://www.moi.gov.tw/files/site_stuff/321/2/year/year.html. (3) Socio-Economic Geographic Information System: https://segis.moi.gov.tw/STAT/Web/Platform/QueryInterface/STAT_QueryInterface.aspx?Type=1.
